# Real-Time Object Tracking with Template Tracking and Foreground Detection Network

**DOI:** 10.3390/s19183945

**Published:** 2019-09-12

**Authors:** Kaiheng Dai, Yuehuan Wang, Qiong Song

**Affiliations:** 1 School of Artificial Intelligence and Automation, Huazhong University of Science and Technology, Wuhan 430074, China; daikaiheng@hust.edu.cn (K.D.); samsong@hust.edu.cn (Q.S.); 2 National Key Lab of Science and Technology on Multi-spectral Information Processing, Wuhan 430074, China

**Keywords:** object tracking, template matching, foreground detection, convolutional neural network

## Abstract

In this paper, we propose a fast and accurate deep network-based object tracking method, which combines feature representation, template tracking and foreground detection into a single framework for robust tracking. The proposed framework consists of a backbone network, which feeds into two parallel networks, TmpNet for template tracking and FgNet for foreground detection. The backbone network is a pre-trained modified VGG network, in which a few parameters need to be fine-tuned for adapting to the tracked object. FgNet is a fully convolutional network to distinguish the foreground from background in a pixel-to-pixel manner. The parameter in TmpNet is the learned channel-wise target template, which initializes in the first frame and performs fast template tracking in the test frames. To enable each component to work closely with each other, we use a multi-task loss to end-to-end train the proposed framework. In online tracking, we combine the score maps from TmpNet and FgNet to find the optimal tracking results. Experimental results on object tracking benchmarks demonstrate that our approach achieves favorable tracking accuracy against the state-of-the-art trackers while running at a real-time speed of 38 fps.

## 1. Introduction

Object tracking is a significant field in computer vision. Given an object in the first frame, the goal of object tracking is to locate the object in the remaining frames. The tracked objects may undergo several difficult challenges, such as scale changes, occlusion, appearance changes of both the object and the scene, camera motion, fast motion, etc., which make object tracking a still challenging task, although there has been a big improvement in recent research. In this paper, we focus on the object representation and object localization to perform robust object tracking.

Recently, in object representation, convolutional neural networks (CNN) are very powerful in extracting discriminative feature representations and have been successfully applied to object tracking. Some Siamese network-based tracking methods [1,2,3,4], which train from scratch in an end-to-end manner, have obtained excellent accuracy with high efficiency; however, unfortunately, they are data-driven trackers and need extensive training data to offline train. In addition, they may fail to track the object they do not see in the training sets. As we know, it is costly to construct a large training dataset that contains all kinds of objects and scenes, which limits their applications. Some deep trackers [5,6,7] use those CNN models pretrained for the classification tasks to extract deep features, because of their powerful representation. Wang et al. [8] and Ma et al. [9] found that the output features of top layer in the pretrained model encode the semantic information of targets, while the features of lower layer can provide more precise localization. Besides, combined deep features from multiple layers also have been applied to recent trackers [10,11,12] and obtained a boosting performance. However, these trackers cannot achieve competitive performance in both speed and accuracy for two reasons. First, these CNN features pretrained for the image classification task are not well coupled to object tracking task since the same kinds of objects can be considered as a target in a sequence and as a background in another. Although fine-tuning pretrained network in the first frame is an optional way to make deep features generic to the tracked objects, it is susceptible to over-fitting due to the lack of training samples. Second, high dimensions of deep features result in heavy computing. To avoid heavy computing, a straightforward way is to compress deep feature representation. However, the compressed features may degrade the overall performance. In object localization, correlation filter (CF) and cross-correlation (CC) are popular in recent works. The CF-based trackers [13,14,15] solve a ridge regression problem efficiently in Fourier domain, which helps achieve high frame rates. DCFnet [16] and CFnet [17] embed a CF layer into deep neural network to perform the correlation tracking process. Recently, more and more Siamese network trackers [1,18,19] utilize cross-correlation to compute the similarity between target template and the search feature maps, for its efficient implementation in convolution layer. However, these methods are still met with high computation burden when the input features’ channels are large.

To address the above issues, in object representation, we adopt a modified VGG model as backbone network and fine-tune the last block parameters to extract generic and specific deep features. Furthermore, we introduce a fast template tracking network (TmpNet) to perform template tracking, which learns channel-wise target template online, reducing the computation burden very efficiently. The motivation is that different feature channels encode different aspect of objects. We make full use of all aspects of feature channels and do not introduce extra computation burden. In addition, a foreground detection network (FgNet) is proposed to improve the tracking accuracy. To ensure all components work closely, we use a multi-task loss to end-to-end train the overall framework, which also helps the extracted feature representation be not only good at object-level template tracking but also good at pixel-wise foreground detection. To improve generalization network performance, an image augmentation method is developed to augment the training samples in the first frame of a test sequence. Compared to our previous work [20], we propose a more discriminative feature representation model, a fast template tracking branch, and a sample augmentation method to process real-time accurate object tracking.

Our main contribution can be summarized as follows:

- We combine feature representation backbone network, TmpNet and FgNet into an end-to-end framework for real-time object tracking.
- In object representation, we adopt a modified pretrained VGG as backbone network where the last block parameters are fine-tuned via a multi-task loss in the first frame of a test sequence, which enhances the discrimination of feature representation.
- In object localization, TmpNet for channel-wise target template tracking and FgNet for fast foreground detection are combined to find the optimal tracking results, boosting the tracking performance.
- We performed comprehensive experiments on four benchmark datasets: OTB2013 [21], OTB-2015 [22], TC128 [23], and UAV123 [24]. Our tracker achieves outstanding performance, operating at 38 fps on a single GPU, while obtaining an AUC of 67.7% on OTB2013, 64.1% on OTB2015, 55.4% on TC128 and 47.1% on UAV123.

## 2. Related Work

Our approach is related to real-time tracking, template tracking, and foreground detection; we give a brief review on these three aspects.

### 2.1. Correlation-Filter Based Real-Time Tracker

In recent years, the correlation filter (CF) based trackers are popular in real-time tracking. Since the CSK tracker [13] introduces correlation-filter to the visual tracking field, several extensions have been proposed to improve the tracking performance for its computational efficiency in frequency domain. Heriques et al. [14] used correlation filters in kernel space with the CSK method, and then improved to KCF tracking algorithm by using HOG features instead illumination intensity features, which helps KCF achieve the state-of-the-art performance. The DSST [25] tracker extends the CF tracker to adapt to scale changes by introducing multi-scale correlation filters. LCT [26] improves the performance of CF tracker in long-term object tracking. BACF [15] proposes a background-aware correlation filter to mitigate the boundary effects of CF tracker in the Fourier domain and exploits a masking matrix to allow search samples larger than the filter, which makes it achieve better performance. STRCF [27] introduces a spatial-temporal regularization to handle boundary effects without much loss in efficiency and achieves superior performance. To make full use of feature channel, ACFN [28] develops an attentional mechanism that chooses a subset of the associated correlation filters to increase robustness and computational efficiency. SCT [29] presents a new attentional feature-based correlation filter that focuses on distinctive attentional features. These trackers are processing in handcrafted features, such as Hog or color, which makes it fast compute using fast Fourier transform; however, it is still costly to calculate with deep features. Recently, some researchers have found ways to calculate CF with deep features quickly. DCFnet [16] treats CF as a special correlation layer embedded in a Siamese network to learn the convolutional features and perform the correlation tracking process simultaneously. CFnet [17] introduces a correlation filter to fully-siamese network to speed up tracking. VDCFnet [30] replaces one traditional convolutional filter with two novel vector convolutional filters. Due to the boundary effect, those trackers cannot obtain accurate results. The Staple [31] tracker, which combines template response and foreground response to improve the tracking performance, is the closest antecedent of our work. However, this approach uses traditional method to obtain template response and foreground mask, especially applied color histogram to predict object pixels, which makes them less robust in handling illumination changes. In this paper, we train a fully convolution network to obtain foreground mask. In addition, a fast template tracking network, which learns channel-wise target template online, is proposed to obtain accurate template response.

### 2.2. Real-Time Template Tracking Methods

In recent years, deep convolutional neural networks (DCNN) demonstrate their superior capabilities in various vision tasks. Some of researchers introduce DCNN to object tracking field with a Siamese network way; they model object tracking task as a similarity learning problem so that they can apply Siamese network architecture to compute the similarity between the target template and the search image in the remaining frames. The Siamese network consists of two branches: one branch encodes the object information, the other encodes the search image information, and then computes their similarity to get the target’s location in the remaining frames. GOTURN [32] adopts a Siamese network as feature extractor and trains a conv-net to directly regress the object in the remaining frames from the object information in the first frame. SiamFC [1] introduces a cross-correlation layer to compute the similarity of the target and the search image, which achieves robust and fast calculations of the similarity of each sub-windows in the search image densely. These trackers achieve real-time tracking, but do not obtain satisfactory performance due to no model update. Thus, Dsiam [3] tracker presents a running average template method to update the tracking model. Memtrack [2] introduces an LSTM as a memory controller to online update template for adapting to the appearance changes. Structsiam [19] proposes a robust structure-based Siamese network to learn local patterns of the object and structure relationships for more accurate object tracking. EAST [33] attempts to speed up the tracker by early stopping the feature extractor if the low-level features are sufficient to track the target. SA-siam [18] proposes a twofold Siamese network, which composes of a semantic branch and an appearance branch and combines their results for robust visual tracking. OSNV [34] proposes an online Siamese network to adapt to target appearance changes. Kalman–Siam [35] combines Kalman filter and multifeature fusion Siamese network for real-time visual tracking. SiamRPN [4] introduces a RPN branch to the Siamese network to obtain accurate bounding box regression. RASNet [36] presents a residual attention and channel attention to Siamese network for adapting the model without updating the model online. DaSiamRPN [37] introduces a novel distractor-aware module to SiamRPN network to perform incremental learning for accurate and long-term tracking. However, these kinds of trackers need extensive training sets to train the network offline. In addition, the learned models are not necessarily reliable for the objects that are not seen in the training sets, which may limits their applied ranges. The UCT [38] and CREST [5] trackers, although they declare they reformulate CF operation as a one-convolution layer, are still template tracking methods in essence. The difference from traditional template tracking methods is they learn the target template in a convolution layer instead of directly cropping it in the first frame. However, CREST cannot achieve real-time tracking duo to heavy computing in residual learning, and UCT cannot obtain better performance because of compressing deep features.

In this paper, although our tracker learns the target template in a convolution layer, our solution is to learn the channel-wise target template using depth-wise convolution, which helps reduce the computational burden. In addition, we try to make full use of deep features by transferring learning the pretrained feature representation with a multi-tasks loss, and the augmented samples are used to learn more discriminative features and enhance the representation of the target.

### 2.3. Foreground Detection Methods

Since fully convolutional networks (FCNs) [39] were introduced to semantic segmentation and showed significant improvement over conventional approaches by a large margin, many extending works have been developed to solve segmentation related problems, including foreground detection problem. Cascade CNN [40] proposes a multi-resolution convolutional neural network with a cascade architecture to detect foreground in moving object videos. FgSegNet [41] uses a triple convolutional neural network for multi-scale feature encoding the foreground segmentation. Although these methods obtain considerable performance in the surveillance videos, there are some problems in introducing them to the object tracking task. First, these approaches are applied in the static camera videos. Second, they need multi-frames to train the network. Third, they take too much time to process detection task. In addition, in object tracking task, we do not pursue pixel-to-pixel precise segmentation, and coarse foreground detection can satisfy our needs to improve the tracking performance. With these considerations in mind, we propose a small FCN network to fast obtain coarse foreground mask. The tracking results demonstrate that it is indeed effective to boost tracking performance without adding too much computation burden.

## 3. Our Method

In this section, we clarify the details of our approach in four separate subsections considering: (1) formulation and motivation; (2) the details of the architecture; (3) the training details; and (4) online tracking processing.

### 3.1. Formulation and Motivation

In the online learning object template method, in the first frame, the extracted backbone network feature of training sample $s_{k}$ and its corresponding label $l_{k}$ that is generated in frame 1 are selected to learn the object template *t* by minimizing the following objective [5]:

(1) $$\min_{t}\sum_{k=1}^{K}L\left(s_{k}⋆t-l_{k}\right)+\lambda R\left(t\right)$$

here $s_{k}\in R^{w_{s}\times h_{s}}$ and $t\in R^{w_{t}\times h_{t}}$ refer to the feature (the output of backbone network) of *k*th training sample and target template, respectively, and *K* is the sum of training samples. $l_{k}\in R^{w_{l}\times h_{l}}$ is the desired corresponding correlation response and $w_{l}=w_{s}-w_{t}+1,h_{l}=h_{s}-h_{t}+1$. λ is a regularization and ☆ is the spatial convolution operator. *L* is a loss function and L2 loss is adopted as usual. A regularization term R(t) is used to limit template complexity and prevent over-fitting. The drawback of Equation (1) is that it only learns the target template. Its feature extractor is a pretrained convolution network and not fine-tuned in the first frame, which make the extracted features not coupled to the tracked object.

Thus, we propose to learn the feature representation $s_{k}$. In addition, a new task, i.e., foreground detection, is proposed to take $s_{k}$ as a variable too, which helps the feature representation be good at not only object-level template tracking but also pixel-wise distinguishing the target from background. All parameters are trained in the first frame of a test sequence. Moreover, to improve the ability of template differentiated from the background and to make full use of each feature channel’s semantic information, we propose to learn channel-wise object template. In general, our new goal is to minimize the following objective:

(2) $$\begin{array}{cc} \min_{s_{k},t,\varpi } & \sum_{k=1}^{K}L_{tmp}\left(\sum_{c=1}^{C}s_{k}^{c}⋆t^{c},l_{k}\right)+\sum_{k=1}^{K}L_{fg}\left(\tau \left(s_{k},\varpi \right),y_{k}\right)\\ \; & +\lambda_{templ}\sum_{c=1}^{C}R\left(t^{c}\right)+\lambda_{fg}R\left(\varpi \right) \end{array}$$

where $s_{k}^{c}$ and $t^{c}$ are the *c*th channel of the *k*th training image’s backbone network feature and *c*th channel of object template, respectively; $y_{k}\in R^{w_{s}\times h_{s}}$ is the foreground mask; and the function τ is a feature decode transformation such that $\tau (s_{k})$ can generate a foreground mask according to the model parameters ϖ. A regularity is added to foreground model R(ϖ) to limit the complexity and avoid over-fitting.

### 3.2. Details of the Overall Architecture

**Figure 1:**
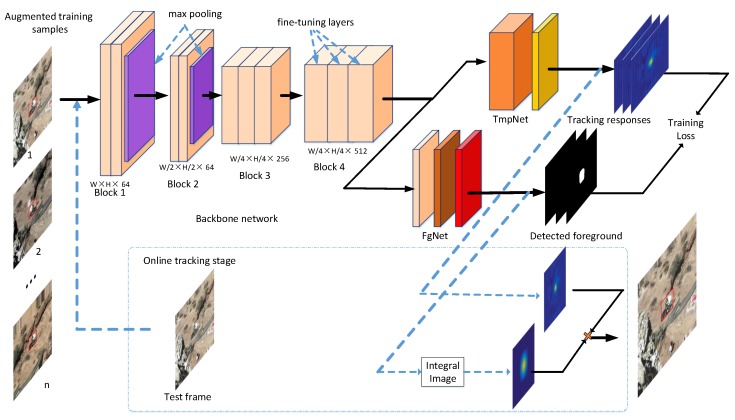
Overall architecture of our TFnet. In the proposed framework, the last block parameters of backbone network, channel-wise target template in the TmpNet and all the parameters in the FgNet are jointly trained with the augmented training samples. The black arrows indicate the training processes, while blue arrows are online tracking stage.

We now detail our network architecture to implement Equation (2). It can be divided into three parts: backbone network, template tracking network, and foreground detection network. The overall architecture is shown in Figure 1.

Backbone network . We start from a pretrained modified VGG-16 network for feature extraction. In the deep network, the stride affects the degree of tracking precision. A big stride results in big jumps in the moving object’s location, while small stride makes the feature representation lack semantic information. Thus, to extract discriminative and semantic feature representation without loss tracking precision, we utilize the first four blocks of VGG-16 by removing the third and fourth max-pooling layers, as illustrated in Figure 1. However, there are still too many parameters in this modified pretrained network. If we train it in the first frame, it will result in over-fitting due to lack of training samples. We observe that there is no need to transfer learning of all the parameters in the pretrained network. Thus, we suggest freezing the pretrained parameters of the first three block; only the fourth block is used for fine-tuning. In this setting, our backbone network can not only inherit the discriminative representation of VGG network but also generate the deep features coupled to the tracked object without over-fitting.

Template tracking network. We adopt a depthwise separable convolution to perform template tracking. The channel-wise object template, which is the parameter in depthwise separable convolution, is initialized in the first frame and performs template tracking in test frames. The depthwise separable convolution decomposes a standard convolution into depthwise convolution and pointwise convolution. Depthwise convolution applies one filter on each channel followed by a 1×1 Pointswise convolution. We leverage this idea, and apply the depthwise convolution to perform fast template tracking. The parameter of depthwise convolution is the learned target template. Its size should cover the object size, and its channel is equal to the input features. With depthwise separable convolution, we can learn object template on each channel independently. More importantly, it helps to reduce the computational cost according to the recent work by et al. [42]. In the proposed framework, the use of depthwise separable convolution immediately reduces the required computation by 20 times compared to the standard convolution.

Foreground detection network. We adopt a simple but effective small FCN network to implement the foreground decode function τ, which consists of three layers: a convolution layer, a deconvolution convolution layer, and a softmax layer. The input features have a large dimension, i.e., 512, which result in large computation to directly compute the foreground mask. To improve computational efficiency and to increase nonlinearity of decision function in our network, we apply a 1×1 convolutional layer to project high channels feature map into a lower dimension. Then, the projected features are operated with a 3×3 deconvolution layer, with a stride of 1 to promise same size output, to decode to 1 feature map. Finally, a softmax function is applied to the last layer to generate a probability mask for each pixel to encode the probability of being a foreground or background pixel by a value that is between 1 and 2.

### 3.3. Training Details

Samples and labels are important in training object tracking network. How to generate and label the samples bothers many researchers. The common method is to label a sample positive/negative based on its distance from the true object location or their intersection-over-union criterion (overlap ratios of a sample position and estimated object region). In this paper, we crop the search images from the center target position, and the template tracking label is generated with a 2D Gaussian function centred around the object. As for the foreground label, it is hard to obtain its precise foreground mask with segmentation methods because the tracked objects in most of tracking sequences may not be a natural object. Thus, we directly apply the given bounding box to generate coarse foreground label; positions inside the bounding box are considered as foreground, while others are regarded as background.

The loss function $L_{tmp}$ is the L2 loss while a cross-entropy loss function is chosen to $L_{fg}$ to learn foreground mask accurately.

(3) $$L_{fg}=-\sum_{k\in Y_{f}}logP\left(y_{k}=1|s_{k};\varpi \right)-\sum_{k\in Y_{b}}logP\left(y_{k}=0|s_{k};\varpi \right)$$

where $y_{k}\in 0,1$, k=1,..., and $Y_{f}$ and $Y_{b}$ are the positive and negative labeled pixels. P(·) is obtained by applying a softmax function to the activation of the final layer.

Due to the lack of training samples, the tracking model will be over-fitted or prone to the background patterns when it needs hundreds of iterations to train. We augment the training samples to alleviate this problem by simulating appearance changes of the tracked object.

**Figure 2:**
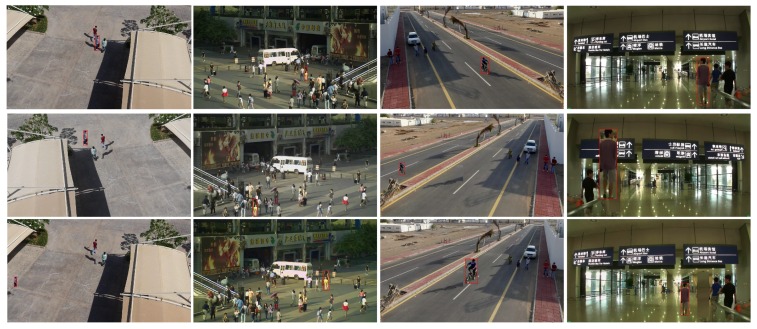
Examples of the augmented images. The first row images are the first frame of a test sequence, while the second and third row are the examples of the augmented images. The tracked objects are marked in a red rectangle.

**Augmentation samples generation strategy.** Since the tracked objects may undergo scale changes, illumination changes, moving, rotation, and occlusion, our image augmentation method should capture all of these aspects, except for the occlusion. This is because the goal of our TmpNet is to learn the object template and occlusion may introduce more background information to the template. We first extract foreground object according to the ground truth bounding box, then remove the foreground object and finally generate its background image via in-painting the cut-out area. Then, we randomly obtain the illumination change factor for background, scale and rotation factor for object, and location factor to generate synthetic data. Here are more details and the examples of augmented images are shown in Figure 2:

(1) Scale changes: We randomly choose a scale factor α∈[0.7,1.5] to change object scale via $\left(w,h\right)=\alpha *\left(w_{o},h_{o}\right)$.
(2) Object rotation: We change object rotation by choosing a rotation angle factor θ, where $\theta \in [-30^{\circ },30^{\circ }]$.
(3) Object flip: Since some objects may go through object flip, we simulate them via flipping an object up and down or left and right.
(4) Illumination change: We change the illumination of image by random to choose saturation *S* and value *V* in HSV space, $\overset{‘}{x}=ax^{b}+c$, where a∈[0.95,1.05], b∈[0.7,1.3], and c∈[−0.07,0.07].

### 3.4. Online Tracking Processing

We combine the scores of template tracking and foreground detection to locate the object accurately. In a new frame, we extract the search images from the center target position predicted in the last frame and feed it into the proposed framework. The object location $p_{t}$ that locates in frame $x_{t}$ is chosen from a combined score map to maximize the score:

(4) $$p_{t}=\arg\max_{p\in S_{t}}\left[\nu_{tmp}f_{tmp}\left(x\right)+\nu_{fg}f_{fg}\left(x\right)\right]$$

where $\nu_{tmp}$ and $\nu_{fg}$ are the cost factors to balance those two models.

The template tracking score is the output of TmpNet, which is computed by a channel-wise object template (depthwise separable convolution) with the input of a *C*-channel feature image $s_{x}$. To perform robust tracking, we adopt two templates to calculate tracking scores: static template $t_{s}$ and adaptive template $t_{a}$. They are both initialized in the first frame. The static template is not updated, while the adaptive template is updated in each frame.

(5) $$f_{temp}\left(x;t_{s},t_{a}\right)=\sum_{c=1}^{C}\left\{t_{s}\left\lfloor c\right\rfloor ⋆s_{x}\left\lfloor c\right\rfloor +t_{a}\left\lfloor c\right\rfloor ⋆s_{x}\left\lfloor c\right\rfloor \right\}$$

The foreground scores are generated with the output masks of FgNet. Different from TmpNet, the output of FgNet has the same size as the input features. However, for effective linear combination, foreground scores and template tracking score maps should have the same size and the magnitudes should be compatible. For those considerations, an all-ones convolution filter that has the same size with the object template is adopted to densely slide the search foreground mask and obtain the integral image; besides, we add a spatial penalty to the foreground scores to constrain its spatial distribution:

(6) $$f_{fg}\left(x;t_{1}\right)=\psi \left(x\right)⊙\left(\frac{1}{R}\left(t_{1}⋆\varpi \left(x\right)\right)\right)$$

where ϖ(x) is the foreground mask. $t_{1}$ is an all-ones convolution filter, which has the same size as the object template. *R* is the sum of template size in feature map, which is used to normalize the score map. ψ(x) is the spatial penalty for giving more weight to the object last appearing and ⊙ is element-wise multiply.

**Algorithm 1:** Tracking with TFnet. 1:**Input:** Frames${\left\{I_{i}\right\}}_{i=1}^{N_{f}}$, initial target bounding box $b_{1}$ 2:Augment the training samples according to the augmentation generation strategy. 3:Learn the variables in Equation (2) with the augmented training samples, where $s_{k}$ corresponding to the backbone network; *t*, the target template; ϖ, the foreground network 4:Copy the target template *t* to an adaptive template $t_{a}$ and static template $t_{s}$ 5:For frame *i* = 2:$N_{f}$ 6:     Extract the search images according to the result in last frame $b_{i-1}$ 7:     Forward propagate the search images and predict the object location with Equation (4) 8:     Generate a new training sample and its corresponding label based on the predicted       results 9:     Update the adaptive target template $t_{a}$ with Equation (7) 10:End 11:**Output:** Tracking results ${\left\{b_{i}\right\}}_{i=2}^{N_{f}}$

**Scale estimation**. We locate the target and estimate its scale simultaneously using a scale pyramid input. When a new frame comes, we extract three scale searching images and feed it into the framework to compute response maps of scale pyramid searching images. The position of the maximum response value is the location of the object, and its corresponding scale is used to estimate a new scale via a linear fusion function.

**Model update**. Updating the overall framework directly results in heavy computing; thus, we present a simple but fast update scheme, in which only the adaptive template is updated. According to the predicted location in the last frame, we generate a new training sample and its corresponding label response map. To avoid double computing, we collect the sample’s feature representation in tracking process stage directly. After that, we feed them into a new learning task to update the adaptive template with the following objective:

(7) $$\arg\min_{t_{a}}∥\sum_{c=1}^{C}s_{k}^{c}⋆t_{a}^{c}-l_{k}{∥}^{2}+\gamma {∥t_{a}∥}^{2}$$

## 4. Experiment

In this section, we first explain the implementation details of our tracker. Then, we present ablation studies of the tracker to evaluate the effectiveness of each component and compare our tracker with the state-of-the-art trackers.

### 4.1. Experiment Setting

In the training stage, we generated the augmented training samples first, and then applied Adam with a learning rate of 5 $\times \;10^{-10}$ to end-to-end train the overall framework. During tracking, we adopted Adam optimizer to update the adaptive template in (7), with a learning rate of 1 $\times \;10^{-12}$ for one iteration in each frame. The pseudo-code for tracking with TFnet can be found in Algorithm 1. We implemented the proposed tracker in Python with the Tensorflow toolbox. All experiments were conducted on a single NVIDIA GeForce GTX Titan X and an Intel(R) Xeon(R)CPU E5-2637 v3 at 3.5 GHz.

### 4.2. Evaluation Benchmarks

OTB2013 [21] and OTB2015 [22]: OTB2013 is the first large datasets to evaluate tracker on both accuracy and robustness, consisting of 50 videos. The images are annotated with ground truth bounding boxes. Besides, each video has different visual attributes, such as illumination variation, scale variation, occlusion, deformation, motion blur, fast motion, in-plane and out-of-plane rotation, out-of-view, background clutter, and low resolution. OTB2015 extends OTB2013 to 100 videos. More videos make it more effective to evaluate trackers.

TC128 [23]: This benchmark consists of 128 color videos to study the role of color information in tracking. Since our tracker needs an RGB-image input, we adopted it to evaluate our tracker. Similar to OTB benchmark, TC128 provides per frame annotated bounding box and 11 videos attributes.

UAV123 [24]: This benchmark is created from an aerial viewpoint. There are 123 sequences captured by unmanned aerial vehicle (UAV) cameras in this dataset. UAV123 has a higher degree of freedom than that of conventional tracking benchmarks, which make it a perfect complement.

### 4.3. Evaluation Methodology

We used the success plot and precision plot to evaluate all trackers. Success metric computes the intersection over union (IOU) of predicted and ground truth bounding box and counts the number of successful frames whose IOU is larger than a given threshold. The success plot shows the ratio of successful frames as IOU threshold is varied from 0 to 1. The precision plot is the percentage of frames in the sequence where the centre location error between predicted and ground truth is smaller than a certain threshold. We present the distance precision values at a threshold of 20 pixels.

### 4.4. Comparisons to State-of-the-Art Trackers

#### 4.4.1. Results on OTB2013

**Figure 3:**
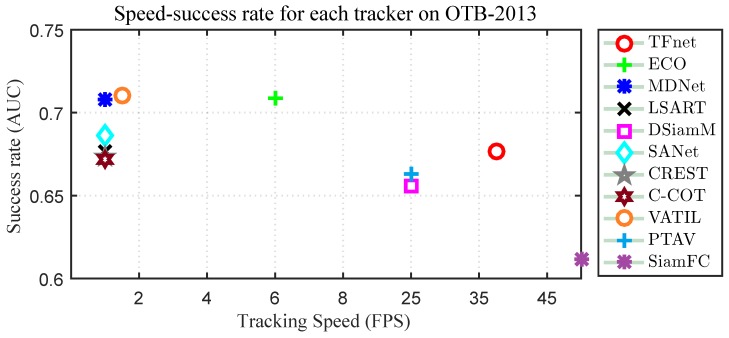
Speed-accuracy plot of state-of-the-art trackers on the OTB2013 dataset.

We evaluated our tracker with state-of-the-art trackers: ECO [11], MDNet [7] , C-COT [10], SANet [43], VITAL [44], LSART [45], DSiamM [3], PTAV [46], CREST [5] and SiamFC [1]. We report their overlap success rates and speed performance in Figure 3. Although some of trackers achieved better performance than our approach, they are not suitable for real-time applications as the tracking speed qA rather slow, only achieving 1–6 frames per second (fps). Our tracker achieved competitive performance and real-time tracking, with 91.3% distance precision rates, 67.7% overlap success rates, and over 38 fps.

**Figure 4:**
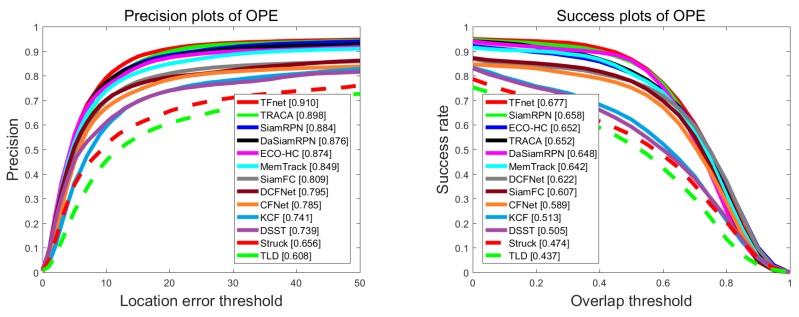
Distance precision plot and overlap success rates plot using one-pass evaluation on the OTB2013 dataset.

We also evaluated the proposed TFnet with comparisons to 13 state-of-the-art real-time trackers: DaSiamRPN [37], SiamRPN [4], Memtrack [2], DCFnet [16], TRACA [6], ECO-HC [11], CFnet [17], SiamFC [1], KCF [14], DSST [25], Struck [47], and TLD [48]. We report the results of success and precision plots using one-pass evaluation. As shown in Figure 4, our tracker achieved state-of-the-art performance among those real-time trackers, with an overlap success score of 67.7% and a distance precision score of 91.0%.

For more details, we show comparisons for five trackers on different attributes in Table 1. Our approach achieved the best results for 7 out of 11 attributes: illumination variation, out-of-plane rotation, scale variation, motion blur, fast motion, in-plane rotation, and background clutters. Moreover, our tracker achieved the best overall performance, 65.9% and 89.9% in success and precision rates, respectively. It demonstrates that our tracker is robust to all kinds of scenes and can adapt to different kinds of challenges in object tracking.

#### 4.4.2. Results on OTB2015

**Figure 5:**
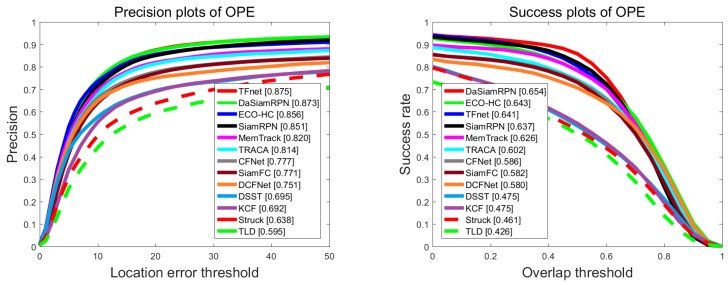
Distance precision plot and overlap success rates plot using one-pass evaluation on the OTB2015 dataset.

Figure 5 illustrates the comparisons of our algorithm with other trackers in both success and precision plots. We can see that our approach still outperformed the other real-time trackers, with 64.3% and 87.5% on overlap success rates and distance precision, respectively.

**Figure 6:**
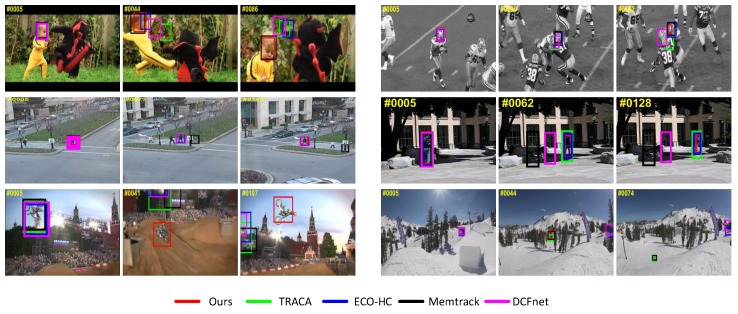
Qualitative tracking results of our tracker, TRACA, ECO-HC, Memtrack, and DCFnet on six challenging sequences. From left to right and top to bottom are Dragonbaby, Football, Human3, Human8, Motorrolling, and Sking, respectively.

We also illustrate qualitative comparisons of our tracker with five state-of-the-art trackers on a subset of videos in Figure 6. The proposed tracker performed as well as Memtrack in fast motion (Dragonbaby) and similar objects (Football,Human3), which demonstrates that our CNN feature representation has outstanding discriminative power compared to the recent Siamese network trackers. The DCFnet and TRACA trackers did not perform well in these two kinds of sequences. This is because the search images of these correlation-based trackers are always multiplied by a hann window to focus on the previous position. Memtrack drifts in Human3 sequence, as Siamese-based trackers always need a template with a large context background to locate the object, which introduces more background information to the tracking template. Besides, most state-of-the-art trackers did not perform well in background clutter and fast motion blurring (Motorrolling), while our tracker could correctly track the targets because we use the augmented training samples to train the framework, which enhances the discrimination of the tracking template; in addition, foreground detection can work well even though objects undergo such difficult challenges. ECO-HC failed to track the object in (Sking), because it uses the conventional feature extraction method, hog, and color. Our tracker performed well in illumination and scale changes (Human8,Sking), which indicates that our model update scheme and scale variation scheme can learn the appearance changes of the object.

#### 4.4.3. Results on TC128

**Figure 7:**
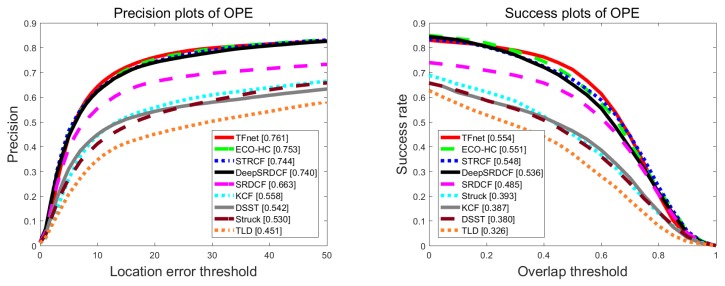
Distance precision plot and overlap success rates plot using one-pass evaluation on the TC128 dataset.

Temple color benchmark contains 128 color image videos. We adopted the same metrics success plot and precision plot to study comparisons of our tracker and six real-time state-of-the-art trackers: ECO-HC [11], STRCF [27], DeepSRDCF [49], SRDCF [50], Struck [47], and KCF [14]. ECO-HC is a real-time variant of ECO based on handcrafted features, Hog and color, while STRCF is a recent state-of-the-art real-time tracker. Figure 7 illustrates the success plot and precision plot of compared trackers. Specifically, our tracker achieved a success score of 55.4% and a precision score of 76.1%, which again surpassed all the compared trackers.

#### 4.4.4. Results on UAV123

**Figure 8:**
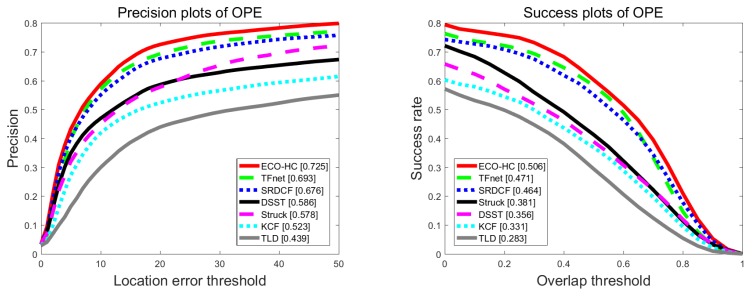
Distance precision plot and overlap success rates plot using one-pass evaluation on the UAV123 dataset.

To further validate the proposed TFnet, we conducted experiments on UAV123. Compared with other tracking benchmarks, UAV123 is from an aerial viewpoint, for which object motion typically has a higher degree of freedom. Figure 8 reports the overall performance of compared trackers. In this figure, we can see that our tracker achieved competitive performance, with a success score of 47.1% and precision score of 69.3%, which is similar to ECO-HC. Possible reasons are that our template tracking is processed with the 1/4 downscale CNN feature maps. Since there are more small size objects in this dataset, our tracker is less effective in handling these kinds of sequences than ECO-HC. As for the attribute of fast motion, our tracker performed better than ECO-HC.

### 4.5. Ablation Study

**Figure 9:**
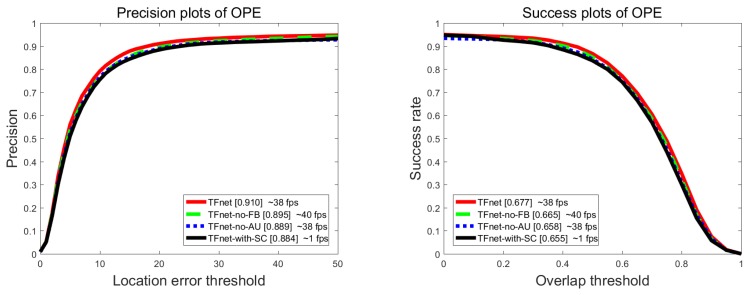
Results of ablation study on the OTB2013 dataset.

To verify the contribution of each component, we conducted ablation analysis to compare the performance of single template tracking branch (TFnet-no-FB), without augmented training samples with the proposed tracker (TFnet-no-AU), and (TFnet-with-SC) that uses a standard convolution (SC) to process template tracking. Figure 9 shows the quantitative evaluation under overlap success (OS) and distance precision (DP). Our tracker could obtain obvious improvement through the integration of foreground detection, which improved OS from 66.5% to 67.7% and DP from 89.5% to 91.0% while only reducing speed from 40 fps to 38 fps. This indicates that, despite exploiting a coarse foreground label to train the foreground detection, our tracker still obtained a boosting tracking performance with low loss in processing time. Besides, using augmented training samples to train the proposed framework obtained a boosted performance on OS (by 1.9%) and DP (by 2.1%). We owe this significant improvement to our data augmentation strategy, which introduces more background information and target appearance changes to learn a more discriminative model. Compared with the baseline TFnet-with-SC, our approach also improved the performances with an OS gain of 2.2% and a DP gain of 2.6% while improving speeds from 1 fps to 38 fps, which demonstrates our TFnet using depthwise separable convolution can not only can speed up tracking processing time but also learn more discriminative object template to improve the tracking performances. In other words, each component in the proposed tracking framework is helpful to improve the performance.

## 5. Conclusions

In this paper, we propose a TFnet for real-time object tracking, which combines feature representation backbone network, channel-wise target template tracking, and foreground detection into a single framework. Template tracking and foreground detection are parallel fully convolution neural networks but share the backbone network. The augmented training samples are used to end-to-end train the overall framework via a multi-task loss to enhance the discriminative of deep features and improve the representation quality of object template. Ablation study experiments showed that each component of our approach is effective to improve the tracking performances. Extensive experiments on OTB2013, OTB2015, TC128, and UAV123 demonstrated that our approach achieves outstanding performance in both accuracy and speed.

References

## Figures and Tables

**Table 1:** Individual attributes comparisons of the proposed method with five state-of-the-art real-time trackers on the OTB2013.

Attributes	Overlap Success Rates on Each Attribute						Distance Precision on Each Attribute					
	TFnet	TRACA	Memtrack	ECO-HC	SiamRPN	DCFnet	TFnet	TRACA	Memtrack	ECO-HC	SiamRPN	DCFnet
IV	**0.659**	0.623	0.588	0.612	0.631	0.596	**0.881**	0.863	0.789	0.793	0.848	0.751
OPR	**0.668**	0.640	0.629	0.632	0.662	0.612	**0.909**	0.897	0.848	0.862	0.890	0.785
SV	**0.672**	0.613	0.654	0.627	0.653	0.619	**0.911**	0.865	0.882	0.838	0.878	0.777
OC	**0.670**	0.644	0.612	**0.670**	0.623	0.645	0.905	0.884	0.810	**0.913**	0.838	0.833
DF	0.675	**0.688**	0.594	0.645	0.677	0.606	0.914	**0.941**	0.797	0.863	0.900	0.787
MB	**0.674**	0.575	0.548	0.610	0.583	0.515	**0.895**	0.771	0.725	0.777	0.786	0.615
FM	**0.671**	0.578	0.585	0.607	0.601	0.534	**0.890**	0.782	0.773	0.797	0.791	0.646
IPR	**0.648**	0.610	0.603	0.589	0.646	0.572	**0.887**	0.859	0.803	0.801	0.867	0.723
OOV	0.686	0.630	0.560	**0.694**	0.615	0.690	0.842	0.743	0.676	**0.883**	0.757	0.820
BC	**0.662**	0.618	0.591	0.606	0.647	0.579	**0.874**	0.844	0.800	0.816	0.868	0.760
LR	0.566	0.470	**0.671**	0.403	0.665	0.429	0.983	0.903	**0.996**	0.750	0.976	0.590
Overall	**0.659**	0.608	0.603	0.609	0.636	0.581	**0.899**	0.850	0.809	0.826	0.854	0.735

The bold fonts indicate the best performance.
